# Alternative Arterial Access Routes for Endovascular Thrombectomy in Patients with Acute Ischemic Stroke: A Study from the MR CLEAN Registry

**DOI:** 10.3390/jcm12093257

**Published:** 2023-05-02

**Authors:** Sabine L. Collette, Elke A. van de Ven, Gert-Jan R. Luijckx, Hester F. Lingsma, Pieter Jan van Doormaal, Adriaan C. G. M. van Es, Ido R. van den Wijngaard, Robert-Jan B. Goldhoorn, Jan Cees de Groot, Wim H. van Zwam, Charles B. L. M. Majoie, Diederik W. J. Dippel, Reinoud P. H. Bokkers, Maarten Uyttenboogaart, on behalf of the MR CLEAN Registry Investigators

**Affiliations:** 1 Department of Radiology, Medical Imaging Center, University Medical Center Groningen, University of Groningen, Hanzeplein 1, Postbus 30001, 9700 RB Groningen, The Netherlands; 2 Department of Neurology, University Medical Center Groningen, University of Groningen, 9700 RB Groningen, The Netherlands; 3 Department of Public Health, Erasmus MC, University Medical Center, 3000 CA Rotterdam, The Netherlands; 4 Department of Radiology and Nuclear Medicine, Erasmus MC, University Medical Center, 3000 CA Rotterdam, The Netherlands; 5 Department of Radiology, Leiden University Medical Center, 2300 RC Leiden, The Netherlands; 6 Department of Neurology, Haaglanden Medical Center, 2501 CK The Hague, The Netherlands; 7 Department of Radiology, Haaglanden Medical Center, 2501 CK The Hague, The Netherlands; 8 Department of Neurology, Cardiovascular Research Institute Maastricht, Maastricht University Medical Center, 6202 AZ Maastricht, The Netherlands; 9 Department of Radiology and Nuclear Medicine, Cardiovascular Research Institute Maastricht, Maastricht University Medical Center, 6202 AZ Maastricht, The Netherlands; 10 Department of Radiology and Nuclear Medicine, Amsterdam University Medical Centers, University of Amsterdam, 1100 DD Amsterdam, The Netherlands; 11 Department of Neurology, Erasmus MC, University Medical Center, 3000 CA Rotterdam, The Netherlands

**Keywords:** alternative access, brachial artery, carotid artery, endovascular thrombectomy, femoral artery, outcome, radial artery, stroke

## Abstract

**Background:** Endovascular thrombectomy (EVT) through femoral access is difficult to perform in some patients with acute ischemic stroke due to challenging vasculature. We compared outcomes of EVT through femoral versus alternative arterial access. **Methods:** In this observational study, we included patients from the MR CLEAN Registry who underwent EVT for acute ischemic stroke in the anterior circulation between 2014 and 2019 in the Netherlands. Patients who underwent EVT through alternative and femoral access were matched on propensity scores in a 1:3 ratio. The primary endpoint was favorable functional outcome (modified Rankin Scale score ≤ 2) at 90 days. Secondary endpoints were early neurologic recovery, mortality, successful intracranial reperfusion and puncture related complications. **Results:** Of the 5197 included patients, 17 patients underwent EVT through alternative access and were matched to 48 patients who underwent EVT through femoral access. Alternative access was obtained through the common carotid artery (n = 15/17) and brachial artery (n = 2/17). Favorable functional outcome was less often observed after EVT through alternative than femoral access (18% versus 27%; aOR, 0.36; 95% CI, 0.05–2.74). The rate of successful intracranial reperfusion was higher for alternative than femoral access (88% versus 58%), although mortality (59% versus 31%) and puncture related complications (29% versus 0%) were more common after alternative access. **Conclusions:** EVT through alternative arterial access is rarely performed in the Netherlands and seems to be associated with worse outcomes than standard femoral access. A next step would be to compare the additional value of EVT through alternative arterial access after failure of femoral access.

## 1. Introduction

Endovascular thrombectomy (EVT) is a highly effective treatment in patients with an acute ischemic stroke due to a large vessel occlusion and is nowadays part of standard care. During EVT, arterial access is usually obtained through the common femoral artery, since it allows for the use of large-lumen devices [1,2,3,4,5,6]. Femoral access may be technically difficult in some patients, for example due to challenging anatomy [7,8]. Failed access may lead to prolonged procedure duration, inability to remove the intracranial thrombus, and eventually to worse patient outcomes [9,10]. In these situations, EVT through an alternative access route may be beneficial. Commonly used alternative approaches are through the radial, common carotid or brachial artery [11,12,13,14,15].

Interest is currently focused on radial access, as it is already widely used in coronary interventions. In the field of interventional cardiology, the radial approach is associated with lower complication rates and higher patient satisfaction than the femoral approach [13,16]. On the other hand, the small vessel diameter and the large amount of vascular anatomical variations may lead to a complex procedure, requiring specific experience of the interventionist [16,17]. Carotid access may lead to a reduced procedure duration because of the shorter distance to the intracranial occlusion and the ability to bypass challenging vasculature (such as tortuous aortic arch or supra-aortic arteries) [12]. Complications related to carotid access can however be severe and may result in poor patient outcomes [12,18]. Brachial access could also result in a shorter procedure duration and an increased patient satisfaction [14]. A drawback of this approach is that serious complications may occur, such as an occlusion of the brachial artery or neuropathy of the median nerve [14,19].

Currently, it is unclear how often an alternative access is used in clinical practice and whether outcomes differ between patients who underwent EVT through alternative and femoral access. This gap in knowledge is due to the high heterogeneity of patients and outcomes that are being studied, lack of large observational studies and unavailability of randomized clinical trials [11,12,13,14,15]. We therefore aimed to investigate the use of alternative access in daily clinical practice and to compare functional, technical and safety outcomes between patients who underwent EVT through alternative access versus femoral access.

## 2. Materials and Methods

### 2.1. Study Design

This was a retrospective observational study from the Multicenter Randomized Clinical Trial of Endovascular Treatment for Acute Ischemic Stroke in the Netherlands (MR CLEAN) Registry, between March 2014 and December 2018. The MR CLEAN Registry is a multicenter, observational study in which all patients who underwent EVT (defined as any procedure with a performed arterial puncture in the angiography suite with the aim to remove the intracranial thrombus) in the Netherlands were enrolled. The study design and patient eligibility criteria have been reported previously [20]. In addition, all local principal investigators from the 17 participating centers of MR CLEAN Registry were requested to identify patients who were not included in the MR CLEAN Registry and had undergone EVT through an alternative access route between January 2015 and October 2019.

The medical ethics committee of the Erasmus University Medical Center Rotterdam in the Netherlands evaluated the study and granted permission to carry out the study as an observational registry (MEC-2014-235). All study protocols and procedures were conducted in accordance with the Declaration of Helsinki and institutional guidelines.

### 2.2. Participants

Patients with an acute ischemic stroke due to a proximal occlusion in the anterior circulation for which they underwent EVT were included. Exclusion criteria were age < 18 year, EVT in a non-MR CLEAN center and inclusion in one of the studies of the Collaboration for New Treatments of Acute Stroke (CONTRAST) consortium [21,22,23].

EVT could be performed through alternative or femoral access. Patients were classified as alternative access in case of successful arterial access through the carotid, brachial or radial artery during a primary or secondary (after failure of femoral access) attempt. Patients were classified as femoral access in case of successful arterial access through the femoral artery during a primary attempt. The choice for alternative access during EVT, the location of the arterial puncture site and the intracranial treatment technique (aspiration, stent retriever, local intra-arterial thrombolytics or a combination of the techniques) was decided by the interventionist.

### 2.3. Data Collection

For the patients included in the MR CLEAN Registry, relevant data were extracted from the MR CLEAN Registry database. The procedural descriptions in the database were reviewed to determine whether EVT was performed through alternative access, and which alternative arterial access route was used. Furthermore, we requested all local principal investigators from the MR CLEAN Registry to identify those patients included in the MR CLEAN Registry who underwent EVT through alternative access. A core lab, blinded to the clinical characteristics and outcome (except for symptom side), evaluated imaging and interventional data. These data were: location of the intracranial occlusion, occlusion side, Alberta Stroke Program Early CT Score (ASPECTS), collateral score, presence, location and type of a concomitant ipsilateral extracranial internal carotid artery lesion (including stenosis ≥ 50%, occlusion and dissection), percutaneous transluminal angioplasty during EVT, carotid artery stenting during EVT and extended Thrombolysis In Cerebral Infarction (eTICI) score before and after EVT. Furthermore, for patients who had experienced a serious adverse event, hospital discharge summaries were reviewed to determine whether the serious adverse event was puncture related.

For patients not included in the MR CLEAN Registry, relevant data (such as alternative arterial access route) were extracted from patient records by the treating physician.

### 2.4. Endpoints

The primary endpoint was favorable functional outcome, which was defined as a modified Rankin Scale (mRS) score ≤ 2 at 90 days after EVT [24].

The secondary endpoints were early neurologic recovery, mortality, successful intracranial reperfusion and puncture related complications. Early neurologic recovery was defined as a National Institutes of Health Stroke Scale (NIHSS) score of 0 or 1, or a decrease of 8 points relative to baseline NIHSS within 24 h after EVT [3]. Mortality was defined as death within 90 days after EVT. Successful intracranial reperfusion was defined as an eTICI score of ≥2B immediately after EVT [25]. Puncture related complications were defined as any untoward medical occurrence or effect causing mortality, a life-threatening situation, a prolonged hospitalization or a persistent significant disability (i.e., any serious adverse event) secondary to the arterial puncture. For patients treated through alternative access, the latter endpoint included puncture related complications due to both the alternative access and the initially performed femoral access attempt.

The procedural variables that were analyzed included time from onset to first arterial puncture attempt and time from first arterial puncture attempt to recanalization. Time from onset to first arterial puncture attempt was defined as the duration from stroke onset to the first arterial puncture attempt in the femoral, radial, carotid or brachial artery in minutes. Time from first arterial puncture attempt to recanalization was defined as the duration of the EVT procedure in minutes, i.e., from the first arterial puncture attempt in the femoral, radial, carotid or brachial artery to the digital subtraction angiography run immediately after EVT.

### 2.5. Statistical Analysis

Baseline characteristics, procedural characteristics and observed outcomes were presented for the groups who underwent EVT through alternative access and femoral access.

Nearest-neighbor propensity score matching was performed to minimize the risk of confounding [26]. First, missing data were imputed using multiple imputation by chained equations based on relevant covariables and outcome. Patients who underwent EVT through alternative access and patients who underwent EVT through femoral access were subsequently matched in a 1:3 ratio. The probability of undergoing EVT through alternative access was calculated for each patient within five imputed datasets averaging the effect estimates, using a logistic regression model in which patients were matched on age, sex, NIHSS score at baseline, affected hemisphere, most proximal occlusion location, medical history of hypertension, hypercholesterolemia and peripheral arterial disease. Matching was performed without replacement, and the caliper width was set to 0.2 SD of the logit for the propensity score [27]. The aforementioned factors were expected to increase the probability of EVT through alternative access for the following reasons. Advanced age, female sex and a medical history of hypertension or hypercholesterolemia seem to increase the risk of vessel abnormalities, such as tortuosity or elongation [28]. In addition, proximal occlusions are more often associated with atherosclerosis and could therefore result in challenging vasculature or peripheral arterial disease [29]. Occlusive peripheral arterial disease may complicate femoral access. For severe strokes, which is reflected through higher NIHSS scores [3], an interventionist may be inclined to make additional efforts to achieve successful reperfusion, such as obtaining alternative access after failure of femoral access. EVT performed through alternative arterial access may be easier for the right hemisphere compared to the left hemisphere.

To facilitate statistical comparisons despite the small number of observations in some of the categories, the mRS score and eTICI score were dichotomized into favorable (mRS ≤ 2) and unfavorable (mRS > 2) functional outcome, and successful (eTICI ≥ 2B) and unsuccessful (eTICI < 2B) intracranial reperfusion, respectively.

The association between alternative arterial access and the primary and secondary endpoints were analyzed using both univariable and multivariable logistic regression analyses. In the multivariable analyses, results were adjusted for time from the first arterial puncture attempt to recanalization. Selection of the covariable was based on the generally known association with stroke outcome.

Data on closure techniques were presented only as raw data for the full cohort. We chose to present the non-imputed data, as imputation led to unrealistic results (after imputation, it seemed that on average 4 closure techniques -including compression- were used per patient). These implausible results were due to the fact that each closure technique had be analyzed and imputed as a separate variable (the closure techniques could not be summarized in one categorical variable, as this would mean that a maximum of one closure technique could have been used per patient).

Two-sided *p* values of <0.05 were considered statistically significant. Data were analyzed using SPSS Statistics 23.0 (IBM Corp., Armonk, NY, USA).

## 3. Results

**Figure 1:**
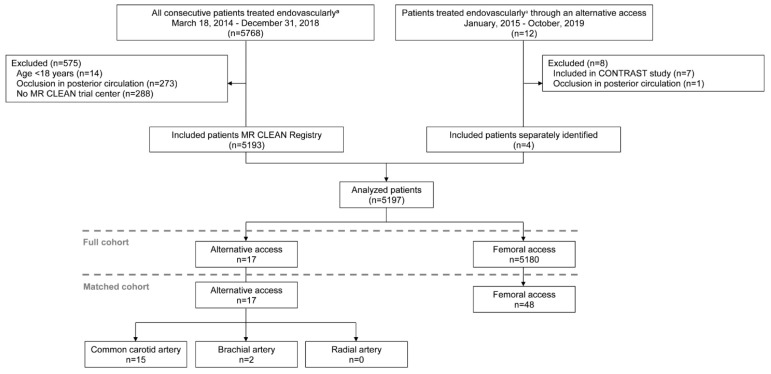
Flowchart of included patients. CONTRAST, Collaboration for New Treatments of Acute Stroke; MR CLEAN, Multicenter Randomized Clinical Trial of Endovascular Treatment for Acute Ischemic Stroke in the Netherlands. n, number. ^a^ Endovascular thrombectomy was defined as any procedure with a performed arterial puncture in the angiography suite with the aim to remove the intracranial thrombus.

A cohort of 5780 patients with acute ischemic stroke who were treated endovascularly between March 2014 and October 2019 was available for this study. These patients were identified in the MR CLEAN Registry database (n = 5768) and, in addition, by the local principal investigators from the MR CLEAN Registry (n = 12). Of the 5768 patients from the MR CLEAN Registry, 5193 patients were eligible for this study (Figure 1). We included 4 of the 12 separately identified patients (patients were excluded due to inclusion in one of the CONTRAST studies [n = 7] and an occlusion in the posterior circulation [n = 1]). These 4 patients were not included in the MR CLEAN Registry because the time from onset to the first arterial puncture attempt was >6.5 h (n = 1, this criterion did not apply to our study) and for unknown reasons (n = 3). In total, 5197 patients met the eligibility criteria of this study. The EVT was performed trough alternative access in 13/5193 patients from the MR CLEAN Registry and in an additional 4 patients treated outside the MR CLEAN Registry. The 17 patients who underwent EVT through alternative access were matched to 48 patients who underwent EVT through femoral access. No matches could be found for one patient treated through alternative access due to a specific combination of baseline characteristics. The alternative access routes consisted of the common carotid artery (n = 15/17) and brachial artery (n = 2/17) (Figure 1).

EVT through alternative access was performed in 5 of the 17 participating EVT centers of the MR CLEAN Registry. In these 5 centers, the number of patients who underwent EVT through alternative access ranged from 1 to 6. The 2 patients who underwent EVT through the brachial artery were treated in the same center.

### 3.1. Baseline and Procedural Characteristics

In the full cohort (before imputation and matching), compared to patients treated with EVT through femoral access, patients treated through alternative access were older, were more likely to have a pre-stroke mRS score > 2, had more frequently an intracranial internal carotid artery or middle cerebral artery occlusion (M3 or M4), were more often treated under general anesthesia, were more likely to have (one of) the puncture site(s) surgically closed, were more often treated with aspiration during EVT and had a longer duration from the first arterial puncture attempt to recanalization (Supplemental Tables S1 and S2). The differences in baseline and procedural characteristics were smaller but still existed in the propensity score matched cohort (Table 1 and Table 2). This finding was consistent with the distributions of the propensity scores and standard deviations of the matched and full cohort (Supplemental Figures S1 and S2).

In most patients (88%, n = 15/17), EVT was performed through an alternative access route if a femoral approach had failed (Table 2). After failure of femoral access, carotid access was obtained because of elongation of the iliac artery or aorta (53%, n = 8/15), occlusion of the femoral or iliac artery (20%, n = 3/15), severe tortuosity of the femoral artery and aorta (6.7%, n = 1/15) or unstable femoral catheter position (reason unknown) (6.7%, n = 1/15). Brachial access was obtained because of elongation of the aortic arch and/or supra-aortic arteries (13%, n = 2/15). The two patients who underwent EVT through primary carotid access had elongated supra-aortic arteries (100%, n = 2/2). The underlying reason for a particular alternative access route was unclear for those patients where several alternative access routes were possible. In both the alternative and femoral access groups, angioseal (85%, n = 11/13, and 94%, n = 2096/2240) was the most frequently used closure technique (Supplemental Table S2).

### 3.2. Outcome Data

#### 3.2.1. Primary Endpoint

**Figure 2:**
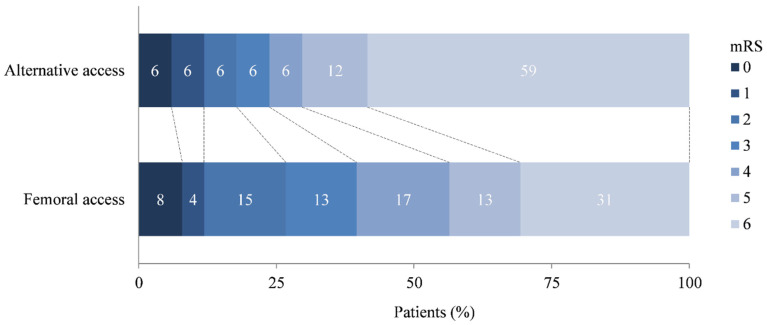
Distribution on the modified Rankin Scale. mRS, modified Rankin Scale.

The rate of favorable functional outcome was lower in patients who underwent EVT through alternative access, albeit not statistically significant (18% versus 27%; odds ratio, 0.44; 95% confidence interval, 0.09–2.25). Also in the multivariable analysis, EVT through alternative access was not associated with favorable functional outcome (adjusted odds ratio, 0.36; 95% confidence interval, 0.05–2.74) (Table 3, Figure 2).

#### 3.2.2. Secondary Endpoints

The proportion of early neurologic recovery seemed almost equal between patients treated though alternative and femoral arterial access (5.9% versus 6.3%). The chance of successful reperfusion was higher (88% versus 58%) after alternative access than femoral access, although mortality (59% versus 31%) and puncture related complications (29% versus 0%) were more common after alternative access. The differences in early neurologic recovery and puncture related complications could not be tested statistically due to the limited number of observations (Table 3).

The puncture related complications included a bleeding from the common carotid artery (n = 2) resulting in a hypovolemic shock (n = 1/2), an atrioventricular block (this was presumably puncture related, however, other causes could not be excluded) (n = 1), an occlusion of the brachial artery (n = 1) and an aneurysm of the brachial artery (n = 1).

In the adjusted analyses, alternative access was not found to be associated with mortality and successful intracranial reperfusion (Table 3).

## 4. Discussion

In this retrospective observational study of the MR CLEAN Registry, EVT through alternative access was rarely performed and only in a few interventional centers. There was no clear association between EVT through alternative access and favorable functional outcome. The rate of successful intracranial reperfusion was higher after alternative than femoral access, although mortality and puncture related complications were more common after alternative access. There was no difference in early neurologic recovery.

EVT through alternative access was particularly performed in a subset of patients of advanced age with anatomically challenging vessels in whom the EVT through femoral access had failed. Compared to the general EVT population [6], the chance of favorable functional outcome was, as expected, lower in patients treated through alternative access (18% versus 46%). This may be due to the fact that, in our study, alternative access was initiated as a rescue treatment after a failed femoral attempt in most patients. The additional effort to obtain vascular access likely prolonged the duration to intracranial recanalization, and consequently decreased the chance of a favorable outcome [6,9]. Furthermore, compared to the general EVT population, patients treated through alternative access may have been more likely to have certain diseases that caused the challenging vasculature (such as connective tissue diseases or chronic hypertension as underlying cause for vessel elongation) [28,30]. The increased vulnerability of the patients treated through alternative access (e.g., due to advanced age or underlying diseases) may have had an impact on outcome.

Most of the literature regarding EVT through alternative access are case reports, case studies and systematic reviews based on observational data (to date, no randomized clinical trials have been conducted) [12,13,18]. Despite the heterogeneous population studied in literature, our study population and outcomes seemed to be relatively consistent with those of other studies on carotid and brachial access [11,12,14]. The rate of favorable functional outcome that was found in our study was almost identical to the rate observed in a systematic review on carotid access (21%) [11]. Another, more recent review [12], showed a slightly higher rate of favorable functional outcome (28%). As both reviews were particularly based on case reports and case studies, which are prone to publication bias and selection bias, the results may have been optimistic. In the aforementioned studies, the outcomes of patients treated through carotid access were not compared to a control group. In a retrospective observational study, the matched control group consisted of patients in whom EVT had been aborted due to failure of femoral access. In that study, carotid access was associated with a shift towards favorable functional outcome. Although the results may have been affected by selection bias, it suggested that EVT through alternative access is a reasonable option for patients in whom femoral access is difficult to obtain [15].

Visual inspection of the mRS score revealed a different distribution between the alternative and femoral access groups. In particular, mortality seemed to be more common among patients treated through alternative access. This could possibly be due to the higher (numerical but not significant) rate of puncture related complications and prolonged procedure duration. We therefore analyzed mortality as a separate endpoint, however, the difference that was observed between both groups could not be confirmed statistically due to low power. As for the other endpoints, the uncertainty of the effect estimate was large, and therefore, definite conclusions could not be drawn. The risk of death after alternative access was comparable between our study and other studies [11,15]. Larger observational studies and systematic reviews and meta-analyses did not compare mortality rates between EVT through alternative and femoral access [11,12,13,15].

Despite the relatively low rate of favorable functional outcome and early neurologic recovery, the chance of successful intracranial reperfusion was, particularly when using alternative access, high. This is a remarkable discrepancy, which may be due to different causes. First, the prolonged duration to recanalization (especially amongst patients treated through alternative access) may have led to expansion of the infarct core and subsequently worse outcomes [6,9,31]. Second, puncture related complications may have had a negative impact on functional outcomes. Third, reperfusion rates may have been overestimated in those patients who were not included in the MR CLEAN Registry due to lack of a core lab assessment [32].

The rate of successful intracranial reperfusion was also high when compared to the general EVT population (71%) [6] or other alternative access populations (65–80%) [11,12,13,14,15]. This may be attributable to the fact that, in this study, there was no protocol for EVT through alternative access. EVT through alternative access may therefore have been performed primarily by experienced interventionists, who were more likely to achieve successful intracranial reperfusion than less experienced interventionists. Moreover, after investing time and taking risks for EVT through alternative access, an interventionist may be inclined to make additional efforts to achieve successful reperfusion.

This study has several limitations. First, despite our best efforts to minimize the risk of bias by matching and adjusting, the results may still have been affected by selection bias. Consequently, patient outcomes may have been overestimated in favor of the alternative access group (e.g., because of treatment by more experienced interventionists), or on the other hand, in favor of the femoral access group (e.g., due to incomplete matching at the expense of the alternative access group). Second, the number of patients treated through alternative access was low, which reduced the precision (and sometimes even hampered the calculation) of the effect estimates, especially in the adjusted analyses. Consequently, some of the results may appear non-significant, while they would have been significant in a larger sample size. Third, we may have missed out on inclusions because not all of the local principal investigators of the MR CLEAN Registry responded to our request to identify patients who had undergone EVT through an alternative access route between January 2015 and October 2019. Also, we may have missed out on patients from the MR CLEAN Registry who were treated through alternative access, as no specific variable existed for the arterial access route. Furthermore, we may have missed out on patients treated through femoral access. As some of the patients treated through alternative access were not included in the MR CLEAN Registry, it is plausible that also a proportion of patients treated through femoral access were not included in the database, while they would have been relevant to our study. Fourth, due to the small sample size, the results could only be adjusted for one covariable. Consequently, our results may have been influenced in unpredictable ways by other potential covariables, such as intracranial treatment technique. In addition, the carotid and brachial approaches could not be analyzed separately, and there were no patients who had undergone EVT through radial access. Hypotheses on the clinical implications per access route could therefore not be formed. Final, due to low power and lack of data, we could not perform any analysis within the subgroup of patients in whom femoral access had failed. Ideally, within this subset of patients, it should be investigated whether EVT through alternative access is superior to termination of EVT. In future prospective studies, this issue should be addressed to provide guidance for clinical practice and to acquire in-depth knowledge on the true effect of EVT through alternative access on patient outcomes.

## 5. Conclusions

EVT through alternative arterial access is rarely performed in the Netherlands and seems to be associated with worse outcomes than standard femoral arterial access. A next step would be to compare the additional value of EVT through alternative arterial access after failure of EVT through femoral access in a randomized setting.

## Figures and Tables

**Table 1:** Baseline characteristics.

Characteristics	Alternative Access (n = 17)	Femoral Access (n = 48)
Age, median (IQR), years	85 (80–89)	84 (80–89)
Female, n (%)	12/17 (71)	37/48 (77)
Medical history, n (%)		
Atrial fibrillation	4/17 (23.5)	17/48 (35.4)
Diabetes mellitus	4/17 (23.5)	6/48 (12.5)
Hypercholesterolemia	7/17 (41.2)	21/48 (43.8)
Hypertension	12/17 (70.6)	37/48 (77.1)
Myocardial infarction	2/17 (11.8)	4/48 (8.3)
Peripheral artery disease	1/17 (5.9)	1/48 (2.1)
Previous stroke	3/17 (17.6)	11/48 (22.9)
Current smoker, n (%)	4/17 (23.5)	8/48 (16.6)
Current medication use, n (%)		
Antiplatelets	8/17 (47.1)	19/48 (39.6)
Anticoagulants	2/17 (11.8)	10/48 (20.8)
Antihypertensives	12/17 (70.6)	39/48 (81.3)
Statins	9/17 (52.9)	21/48 (43.8)
Pre-stroke mRS score > 2, n (%)	6/17 (35.3)	13/48 (27.1)
NIHSS score, median (IQR)	16 (11–19)	14 (9–19)
Location occlusion on CTA, n (%)		
Left hemisphere	10/17 (58.8)	25/48 (52.1)
Intracranial ICA	6/17 (35.3)	14/48 (29.2)
M1	9/17 (52.9)	31/48 (64.6)
M2	1/17 (5.9)	3/48 (6.3)
M3/M4	1/17 (5.9)	0/48 (0)
ICA lesion, n (%)		
Stenosis ≥ 50%	1/17 (5.9)	4/48 (8.3)
Occlusion	1/17 (5.9)	5/48 (10.4)
Dissection	1/17 (5.9)	0/48 (0)
ASPECTS, median (IQR)	9 (8–10)	10 (8–10)
Collateral filling, n (%)		
Absent collaterals	0/17 (0)	3/48 (6.3)
<50% of occluded territory	10/17 (58.8)	15/48 (31.3)
50–99% of occluded territory	4/17 (23.5)	22/48 (45.8)
100% of occluded territory	3/17 (17.6)	8/48 (16.7)
Intravenous thrombolysis, n (%)	13/17 (76.5)	33/48 (68.8)

ASPECTS, Alberta Stroke Program Early CT Score; CTA, computed tomography angiography; ICA, internal carotid artery; M1, middle cerebral artery, first segment; M2, middle cerebral artery, second segment; M3, middle cerebral artery, third segment; M4, middle cerebral artery, fourth segment; mRS, modified Rankin Scale; NIHSS, National Institutes of Health Stroke Scale. IQR, interquartile range; n, number.

**Table 2:** Procedural variables.

Characteristics	Alternative Access	Femoral Access
	**(n = 17)**	**(n = 48)**
General anesthesia, n (%)	10/17 (58.8)	8/48 (16.7)
Alternative access, secondary attempt, n (%)	15/17 (88.2)	N/A
Intracranial treatment, n (%) EVT technique, n (%) ^a^	16/17 (94.1)	42/48 (87.5)
Stent retriever	9/17 (52.9)	36/48 (75.0)
Aspiration	15/17 (88.2)	22/48 (45.8)
Intra-arterial thrombolytics	0/17 (0)	2/48 (4.2)
Carotid artery PTA, n (%)	2/17 (11.8)	4/48 (8.3)
Carotid artery stenting, n (%)	2/17 (11.8)	4/48 (8.3)
Total revascularization attempts, n (%)		
1	4/17 (23.5)	22/48 (45.8)
2	5/17 (29.4)	11/48 (22.9)
3	5/17 (29.4)	10/48 (20.8)
≥4	3/17 (17.6)	5/48 (10.4)
Time from onset to first arterial puncture attempt, median	197 (154–302)	196 (160–254)
(IQR), minutes ^b^		
Time from first arterial puncture attempt to recanalization, median (IQR), minutes ^c^	85 (53–131)	60 (46–84)
Primary alternative access	51 (42–51)	N/A
Secondary alternative access	110 (82–150)	N/A

EVT, endovascular thrombectomy; N/A, not applicable; PTA, percutaneous transluminal angioplasty. IQR, interquartile range; n, number. ^a^ Sum may exceed 100% due to a combination of techniques. ^b^ Time from onset to first arterial puncture attempt was defined as the duration from stroke onset to the first arterial puncture attempt in the femoral, radial, carotid or brachial artery in minutes. ^c^ Time from first arterial puncture attempt to recanalization was defined as the duration of the EVT procedure in minutes, i.e., from the first arterial puncture attempt in the femoral, radial, carotid or brachial artery to the digital subtraction angiography run immediately after EVT.

**Table 3:** Patient outcomes.

Endpoint	Alternative Access (n = 17)	Femoral Access (n = 48)	OR	95% CI	aOR ^a^	95% CI
Favorable functional outcome, n (%) ^b^	3/17 (17.6)	13/48 (27.1)	0.44	0.09–2.25	0.36	0.05–2.74
Early neurologic recovery, n (%) ^c,d^	1/17 (5.9)	3/48 (6.3)	-	-	-	-
Mortality, n (%) ^e^	10/17 (58.8)	15/48 (31.3)	3.18	0.97–10.41	3.16	0.91–11.03
Successful intracranial reperfusion, n (%) ^f^	15/17 (88.2)	28/48 (58.3)	5.34	0.73–38.89	8.62	0.93–80.04
Puncture related complications, n (%) ^d,g^	5/17 (29.4)	0/48 (0)	-	-	-	-

(a)OR, (adjusted) odds ratio; CI, confidence interval; n, number. ^a^ Results were adjusted for time from first arterial puncture attempt to recanalization. ^b^ Favorable functional outcome was defined as a modified Rankin Scale score ≤ 2 at 90 days after endovascular thrombectomy. ^c^ Early neurologic recovery was defined as National Institutes of Health Stroke Scale score of 0 or 1 within 24 h postprocedural, or a decrease of 8 points relative to baseline. ^d^ The effect estimates could not be calculated due to limited numbers of observations. ^e^ Mortality was defined as death within 90 days after endovascular thrombectomy. ^f^ Successful intracranial reperfusion was defined as an extended Thrombolysis In Cerebral Infarction score of ≥2B immediately after endovascular thrombectomy. ^g^ Puncture related complications were defined as any untoward medical occurrence or effect causing mortality, a life-threatening situation, prolonged hospitalization or persistent significant disability (i.e., any serious adverse event) secondary to the arterial puncture.

## Data Availability

Data will not be made available to other researchers as no patient approval has been obtained for sharing data. Syntax files (i.e., documents with commands that execute the SPSS procedure) will be made available from the corresponding author on reasonable request.

## Supplementary Materials

The following supporting information can be downloaded at: https://www.mdpi.com/article/10.3390/jcm12093257/s1, Figure S1: Flowchart of included patients; Figure S2: Distribution of the propensity score before and after matching; Figure S3: Distribution of the standard deviation before and after matching; Table S1: Baseline characteristics full cohort; Table S2: Procedural variables full cohort; Supplemental File: MR CLEAN Registry Investigators—group authors.
