# Supplementary material for: Alternative Arterial Access Routes for Endovascular Thrombectomy in Patients with Acute Ischemic Stroke: A Study from the MR CLEAN Registry
Source: J Clin Med. 2023 May 2;12(9):3257. doi: 10.3390/jcm12093257 (PMC10179212; doi:10.3390/jcm12093257)
Supplement: Supplementary file 1 [file jcm-12-03257-s001.zip › jcm-2294415-supplementary.pdf]

# Supplemental material

## ALTERNATIVE ARTERIAL ACCESS ROUTES FOR ENDOVASCULAR THROMBECTOMY IN PATIENTS WITH ACUTE ISCHEMIC STROKE: A STUDY FROM THE MR CLEAN REGISTRY

Sabine L. Collette, Elke A. van de Ven, Gert-Jan R. Luijckx, Hester F. Lingsma, Pieter Jan van Doormaal, Adriaan C.G.M. van Es, Ido R. van den Wijngaard, Robert-Jan B. Goldhoorn, Jan Cees de Groot, Wim H. van Zwam, Charles B.L.M. Majoie, Diederik W.J. Dippel, Reinoud P.H. Bokkers, Maarten Uyttenboogaart; on behalf of the MR CLEAN Registry investigators.

**Figure S1.** Flowchart of included patients

**Figure S2.** Distribution of the propensity score before and after matching

**Figure S3.** Distribution of the standard deviation before and after matching

**Table S1.** Baseline characteristics full cohort

**Table S2.** Procedural variables full cohort

**Supplemental File.** MR CLEAN Registry Investigators – group authors

**Figure S1.** Flowchart of included patients

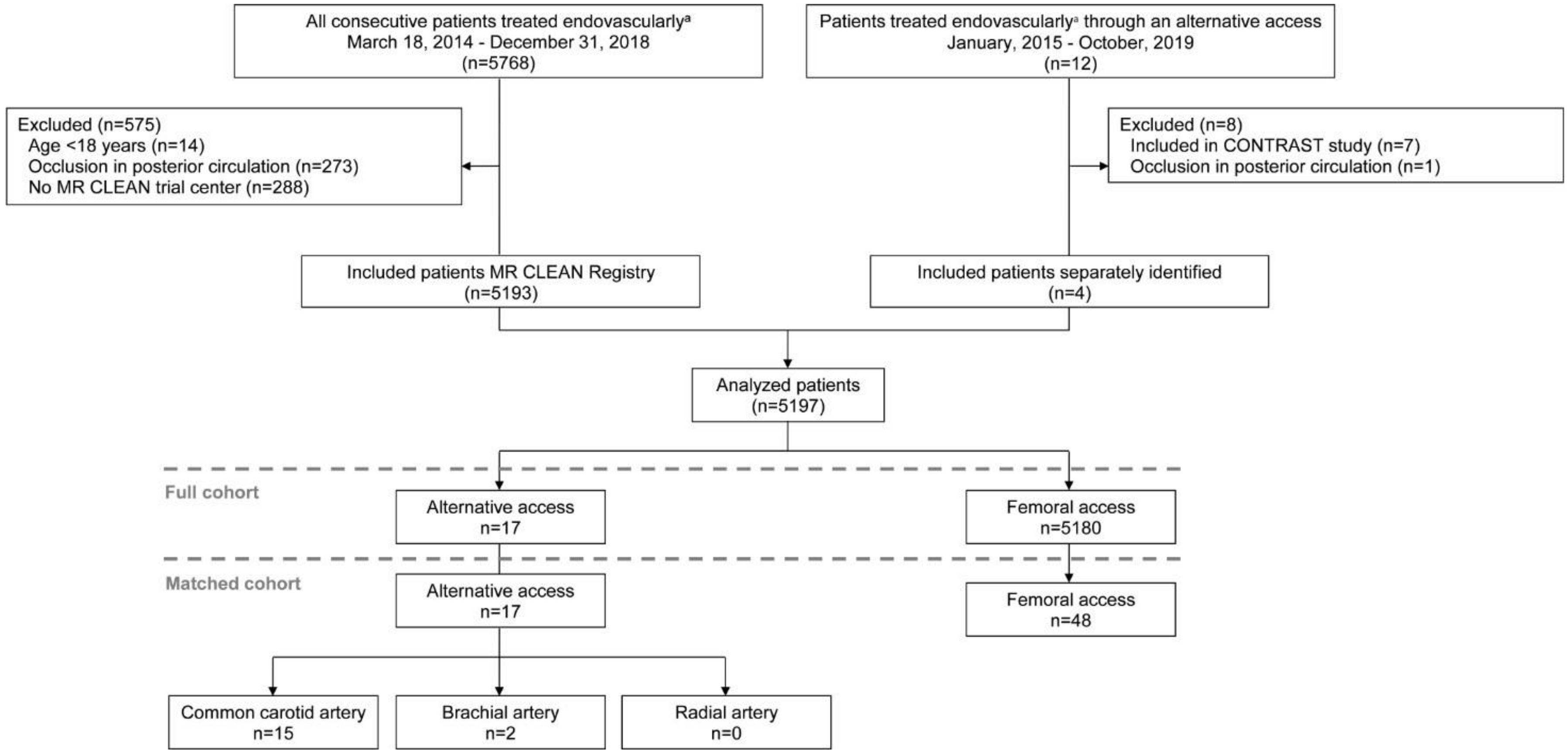


CONTRAST, Collaboration for New Treatments of Acute Stroke; MR CLEAN, Multicenter Randomized Clinical Trial of Endovascular Treatment for Acute Ischemic Stroke in the Netherlands.

n, number.

[a]Endovascular thrombectomy was defined as any procedure with a performed arterial puncture in the angiography suite with the aim to remove the intracranial occlusion.

**Figure S2.** Distribution of the propensity score before and after matching

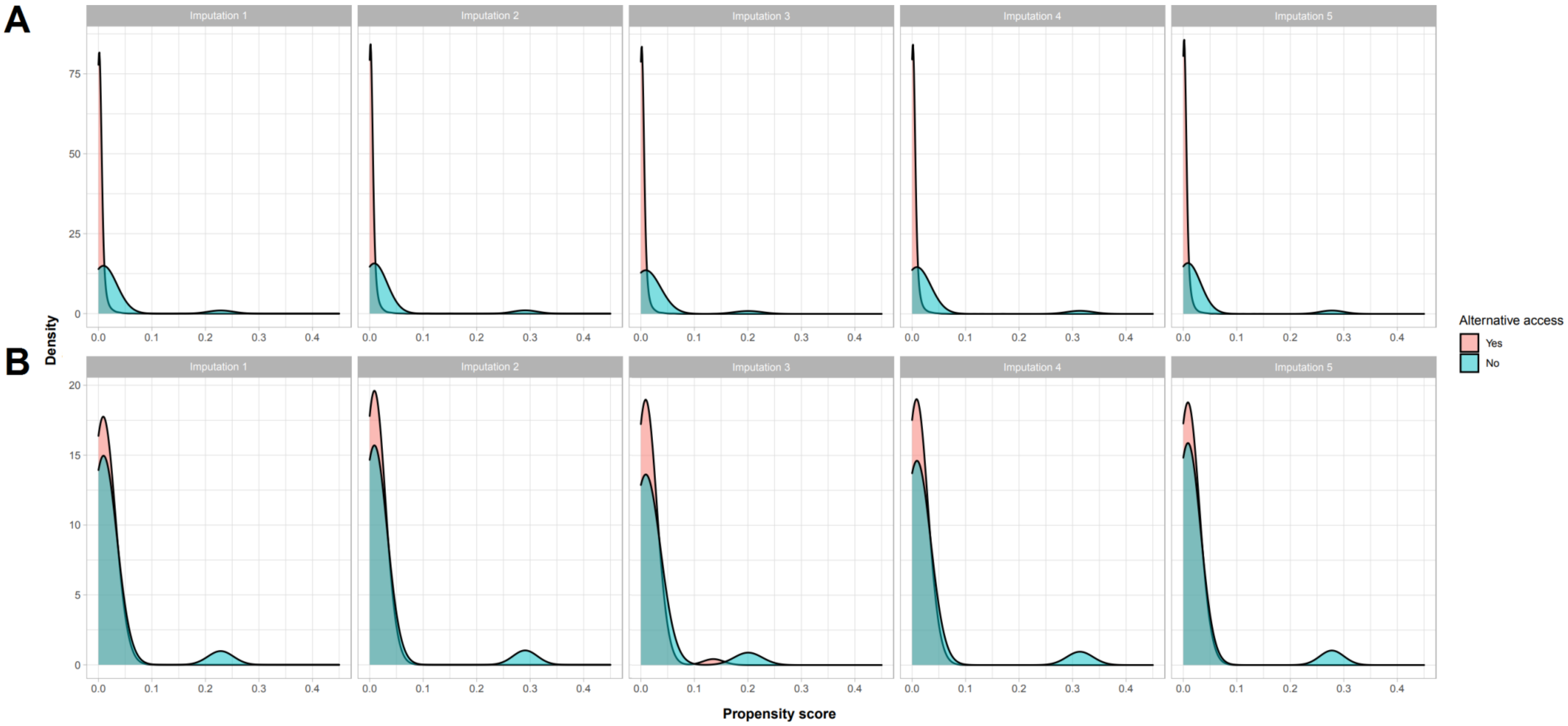


The distribution of the propensity score is displayed on a different scale for the (**A**) full cohort and (**B**) matched cohort to increase readability.

**Figure S3.** Distribution of the standard deviation before and after matching

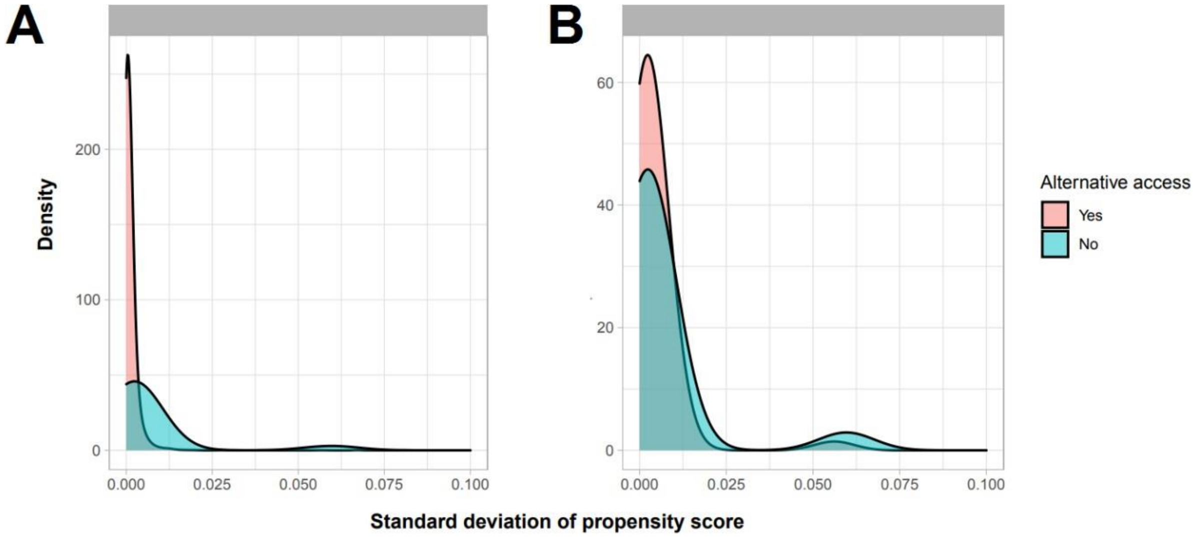


The distribution of the standard deviation of the propensity score is displayed on a different scale for the (**A**) full cohort and (**B**) matched cohort to increase readability.

**Table S1.** Baseline characteristics full cohort

| Characteristics | Alternative access (n=17) | Femoral access (n=5180) | *P* value |
|---|---|---|---|
| Age, *median (IQR)*, years | 85 (80-89) | 72 (63-81) | <.001 |
| Female, *n (%)* | 12/17 (71) | 2479/5180 (48) | .06 |
| Medical history, *n (%)* | | | |
| Atrial fibrillation | 3/16 (18.8) | 1227/5112 (24.0) | .78 |
| Diabetes mellitus | 4/17 (23.5) | 868/5149 (16.9) | .51 |
| Hypercholesterolemia | 7/17 (41.2) | 1541/4972 (31.0) | .37 |
| Hypertension | 12/17 (70.6) | 2706/5085 (53.2) | .15 |
| Myocardial infarction | 2/15 (13.3) | 726/5083 (14.3) | >.99 |
| Peripheral artery disease | 1/17 (5.9) | 466/5084 (9.2) | >.99 |
| Previous stroke | 3/15 (20.0) | 917/5141 (17.8) | .83 |
| Current smoker, *n (%)* | 3/9 (33.3) | 1078/3892 (27.7) | .72 |
| Current medication use, *n (%)* | | | |
| Antiplatelets | 8/15 (53.3) | 1593/5116 (31.1) | .90 |
| Anticoagulants | 2/15 (13.3) | 1009/5149 (19.6) | .75 |
| Antihypertensives | 10/15 (66.7) | 2809/5080 (55.3) | .38 |
| Statins | 8/15 (53.3) | 1826/5069 (36.0) | .18 |
| Pre-stroke mRS score >2, *n (%)* | 6/14 (42.9) | 655/5041 (13.0) | <.01 |
| NIHSS score, *median (IQR)* | 16 (11-19) | 15 (10-19) | .91 |
| Location occlusion on CTA, *n (%)* | | | |
| Left hemisphere | 10/17 (58.8) | 1742/3272 (53.2) | .87 |
| Intracranial ICA | 6/17 (35.3) | 1218/4957 (24.6) | <.01 |
| M1 | 9/17 (52.9) | 2774/4957 (56.0) | |
| M2 | 1/17 (5.9) | 940/4957 (19.0) | |
| M3/M4 | 1/17 (5.9) | 9/4957 (0.2) | |
| A1 | 0/17 (0) | 7/4957 (0.1) | |
| A2 | 0/17 (0) | 9/4957 (0.2) | |
| ICA lesion, *n (%)* | | | |
| Stenosis ≥50% | 0/13 (0) | 431/5180 (8.3) | .62 |
| Occlusion | 0/13 (0) | 492/5180 (9.5) | .63 |
| Dissection | 0/12 (0) | 79/4495 (1.8) | >.99 |
| ASPECTS, *median (IQR)* | 9 (8-10) | 9 (8-10) | .60 |
| Collateral filling, *n (%)* | | | .11 |
| Absent collaterals | 0/14 (0) | 258/4891 (5.3) | |
| <50% of occluded territory | 9/14 (64.3) | 1750/4891 (35.8) | |
| 50-99% of occluded territory | 2/14 (14.3) | 1890/4891 (38.6) | |
| 100% of occluded territory | 3/14 (21.4) | 993/4891 (20.3) | |
| Intravenous thrombolysis, *n (%)* | 11/15 (73.3) | 3575/5150 (69.4) | >.99 |

A1, anterior cerebral artery, first segment; A2, anterior cerebral artery, second segment; ASPECTS, Alberta Stroke Program Early CT Score; CTA, computed tomography angiography; ICA, internal carotid artery; M1, middle cerebral artery, first segment; M2,

middle cerebral artery, second segment; M3, middle cerebral artery, third segment; M4, middle cerebral artery, fourth segment; mRS, modified Rankin Scale; NIHSS, National Institutes of Health Stroke Scale.

IQR, interquartile range; n, number.

**Table S2.** Procedural variables full cohort

| Characteristics | Alternative access (n=17) | Femoral access (n=5180) | *P* value |
|---|---|---|---|
| General anesthesia, n *(%)* | 9/14 (64.3) | 1149/4888 (23.5) | <.01 |
| Alternative access, secondary attempt, *n (%)* | 14/16 (87.5) | *N/A* | |
| Closure technique, *n (%)*[a,b] | | | |
| Angioseal | 11/13 (84.6) | 2096/2240 (93.6) | .20 |
| Mynx | 0/13 (0) | 131/2240 (5.8) | >.99 |
| Starclose | 0/13 (0) | 8/2240 (0.4) | >.99 |
| Exoseal | 0/13 (0) | 1/2240 (0.04) | >.99 |
| Surgical closure | 2/13 (15.4) | 4/2240 (0.2) | <.01 |
| Compression | 1/13 (7.7) | 45/2240 (2.0) | .24 |
| Intracranial treatment, *n (%)* | 16/17 (94.1) | 4409/5180 (85.1) | .72 |
| EVT technique, *n (%)*[a] | | | |
| Stent retriever | 7/13 (53.8) | 3112/4212 (73.9) | .12 |
| Aspiration | 11/13 (84.6) | 1962/4212 (46.6) | <.01 |
| Intra-arterial thrombolytics | 0/13 (0) | 94/4147 (2.3) | >.99 |
| Carotid artery PTA, *n (%)* | 0/9 (0) | 287/4319 (6.6) | >.99 |
| Carotid artery stenting, *n (%)* | 0/12 (0) | 302/4770 (6.3) | >.99 |
| Total revascularization attempts, *n (%)* | | | .36 |
| 1 | 2/11 (18.2) | 1536/3784 (40.6) | |
| 2 | 4/11 (36.4) | 926/3784 (24.5) | |
| 3 | 3/11 (27.3) | 554/3784 (14.6) | |
| ≥4 | 2/11 (18.2) | 768/3784 (20.3) | |
| Time from onset to first arterial puncture attempt, *median (IQR)*, minutes[c] | 195 (150-255) | 195 (148-265) | .87 |
| Time from first arterial puncture attempt to recanalization, *median (IQR)*, minutes[d] | 90 (58-136) | 55 (35-80) | <.01 |

EVT, endovascular thrombectomy; N/A, not applicable; PTA, percutaneous transluminal angioplasty.

IQR, interquartile range; n, number.

[a]Sum may exceed 100% due to a combination of techniques.

[b]The variable included all closure techniques during EVT (i.e., for both alternative and femoral access).

[c]Time from onset to first arterial puncture attempt was defined as the duration from stroke onset to the first arterial puncture attempt in the femoral, radial, carotid or brachial artery in minutes.

[d]Time from first arterial puncture attempt to recanalization was defined as the duration of the EVT procedure in minutes, i.e., from the first arterial puncture attempt in the femoral, radial, carotid or brachial artery to the digital subtraction angiography run immediately after EVT.

**Supplemental Appendix.** MR CLEAN Registry Investigators – group authors

**Executive committee**

Diederik W.J. Dippel[1]; Aad van der Lugt[2]; Charles B.L.M. Majoie[3]; Yvo B.W.E.M. Roos[4]; Robert J. van Oostenbrugge[5,44]; Wim H. van Zwam[6,44]; Jelis Boiten[14]; Jan Albert Vos[8].

**Study coordinators**

Ivo G.H. Jansen[3]; Maxim J.H.L. Mulder[1,2]; Robert- Jan B. Goldhoorn[5,6,44]; Kars C.J. Compagne[2]; Manon Kappelhof[3]; Josje Brouwer[4]; Sanne J. den Hartog[1,2,40]; Wouter H. Hinsenveld [5,6]; Lotte van den Heuvel[1,40].

**Local principal investigators**

Diederik W.J. Dippel[1]; Bob Roozenbeek[1]; Aad van der Lugt[2]; Pieter Jan van Doormaal[2], Charles B.L.M. Majoie[3]; Yvo B.W.E.M. Roos[4]; Bart J. Emmer[3]; Jonathan M. Coutinho[4]; Wouter J. Schonewille[7]; Jan Albert Vos[8]; Marieke J.H. Wermer[9]; Marianne A.A. van Walderveen[10]; Adriaan C.G.M. van Es[10]; Julie Staals[5,44]; Robert J. van Oostenbrugge[5,44]; Wim H. van Zwam[6,44]; Jeannette Hofmeijer[11]; Jasper M. Martens[12]; Geert J. Lycklama à Nijeholt[13]; Jelis Boiten[14]; Sebastiaan F. de Bruijn[15]; Lukas C. van Dijk[16]; H. Bart van der Worp[17]; Rob H. Lo[18]; Ewoud J. van Dijk[19]; Hieronymus D. Boogaarts[20]; J. de Vries[22]; Paul L.M. de Kort[21]; Julia van Tuijl[21]; Issam Boukrab[26]; Jo P. Peluso[26]; Puck Fransen[22]; Jan S.P. van den Berg[22]; Heleen M. den Hertog[22]; Boudewijn A.A.M. van Hasselt[23]; Leo A.M. Aerden[24]; René J. Dallinga[25]; Maarten Uyttenboogaart[28]; Omid Eschgi[29]; Reinoud P.H. Bokkers[29]; Tobien H.C.M.L. Schreuder[30]; Roel J.J. Heijboer[31]; Koos Keizer[32]; Rob A.R. Gons[32]; Lonneke S.F. Yo[33]; Emiel J.C. Sturm[35,47], Tomas Bulut[35]; Paul J.A.M. Brouwers[34]; Anouk D. Rozeman[42]; Otto Elgersma[41], Michel J.M. Remmers[43]; Thijs E.A.M. de Jong[46].

**Imaging assessment committee**

Charles B.L.M. Majoie[3](chair); Wim H. van Zwam[6,44]; Aad van der Lugt[2]; Geert J. Lycklama à Nijeholt[13]; Marianne A.A. van Walderveen[10]; Marieke E.S. Sprengers[3]; Sjoerd F.M. Jenniskens[27]; René van den Berg[3]; Albert J. Yoo[38]; Ludo F.M. Beenen[3]; Alida A. Postma[6.45]; Stefan D. Roosendaal[3]; Bas F.W. van der Kallen[13]; Ido R. van den Wijngaard[13]; Adriaan C.G.M. van Es[10]; Bart J. Emmer[,3]; Jasper M. Martens[12]; Lonneke S.F. Yo[33]; Jan Albert Vos[8]; Joost Bot[36]; Pieter-Jan van Doormaal[2]; Anton Meijer[27]; Elyas Ghariq[13]; Reinoud P.H. Bokkers[29]; Marc P. van Proosdij[37]; G. Menno Krietemeijer[33]; Jo P. Peluso[26]; Hieronymus D. Boogaarts[20]; Rob Lo[18];Wouter Dinkelaar[41]; Auke P.A. Appelman[29]; Bas Hammer[16]; Sjoert Pegge[27]; Anouk van der Hoorn[29]; Saman Vinke[20]; Sandra Cornelissen[2]; Christiaan van der Leij[6]; Rutger Brans[6]; Jeanette Bakker[41]; Maarten Uyttenboogaart[28]; Miou Koopman[3]; Lucas Smagge[2]; Olvert A. Berkhemer[1,3,6]; Jeroen Markenstein[3]; Eef Hendriks[3]; Patrick Brouwer[10]; Dick Gerrits[35].

**Writing committee**

Diederik W.J. Dippel[1](chair); Aad van der Lugt[2]; Charles B.L.M. Majoie[3]; Yvo B.W.E.M. Roos[4]; Robert J. van Oostenbrugge[5,44]; Wim H. van Zwam[6,44]; Geert J. Lycklama à Nijeholt[13]; Jelis Boiten[14]; Jan Albert Vos[8]; Wouter J. Schonewille[7]; Jeannette Hofmeijer[11]; Jasper M. Martens[12]; H. Bart van der Worp[17]; Rob H. Lo[18].

**Adverse event committee**

Robert J. van Oostenbrugge[5,44](chair); Jeannette Hofmeijer[11]; H. Zwenneke Flach[23].

**Trial methodologist**

Hester F. Lingsma[40].

**Research nurses / local trial coordinators**

Naziha el Ghannouti[1]; Martin Sterrenberg[1]; Wilma Pellikaan[7]; Rita Sprengers[4]; Marjan Elfrink[11]; Michelle Simons[11]; Marjolein Vossers[12]; Joke de Meris[14]; Tamara Vermeulen[14]; Annet Geerlings[19]; Gina van Vemde[22]; Tiny Simons[30]; Gert Messchendorp[28]; Nynke Nicolaij[28]; Hester Bongenaar[32]; Karin Bodde[24]; Sandra Kleijn[34]; Jasmijn Lodico[34]; Hanneke Droste[34]; Maureen Wollaert[5]; Sabrina Verheesen[5]; D. Jeurrissen[5]; Erna Bos[9]; Yvonne Drabbe[15]; Michelle Sandiman[15]; Nicoline Aaldering[11]; Berber Zweedijk[17]; Jocova Vervoort[21]; Eva Ponjee[22]; Sharon Romviel[19]; Karin Kanselaar[19]; Denn Barning[10] ; Laurine van der Steen[3].

**PhD / Medical students**

Esmee Venema[40]; Vicky Chalos[1,40]; Ralph R. Geuskens[3]; Tim van Straaten[19]; Saliha Ergezen[1]; Roger R.M. Harmsma[1]; Daan Muijres[1]; Anouk de Jong[1]; Olvert A. Berkhemer[1,3,6]; Anna M.M. Boers[3,39]; J. Huguet[3]; P.F.C. Groot[3]; Marieke A. Mens[3]; Katinka R. van Kranendonk[3]; Kilian M. Treurniet[3]; Manon L. Tolhuisen[3,39]; Heitor Alves[3]; Annick J. Weterings[3]; Eleonora L.F. Kirkels[3]; Eva J.H.F. Voogd[11]; Lieve M. Schupp[3]; Sabine L. Collette[28,29]; Adrien E.D. Groot[4]; Natalie E. LeCouffe[4]; Praneeta R. Konduri[39]; Haryadi Prasetya[39]; Nerea Arrarte-Terreros[39]; Lucas A. Ramos[39] ; Nikki Boodt[1,2,40]; Anne F.A.V Pirson[5]; Agnetha A.E. Bruggeman[3]; Nadinda A.M. van der Ende [1,2], Rabia Deniz[3], Susanne G.H. Olthuis[5,44], Floor Pinckaers[6,44].

**List of affiliations**

Department of Neurology[1], Radiology[2], Public Health[40], Erasmus MC University Medical Center;

Department of Radiology and Nuclear Medicine[3], Neurology[4], Biomedical Engineering & Physics[39], Amsterdam UMC, location University of Amsterdam;

Department of Neurology[5], Radiology & Nuclear Medicine[6], Maastricht University Medical Center+; School for Cardiovascular Diseases Maastricht (CARIM)[44]; and MHeNs School for Mental Health and Neuroscience, Maastricht, the Netherlands[45];

Department of Neurology[7], Radiology[8], Sint Antonius Hospital, Nieuwegein;

Department of Neurology[9], Radiology[10], Leiden University Medical Center;

Department of Neurology[11], Radiology[12], Rijnstate Hospital, Arnhem;

Department of Radiology[13], Neurology[14], Haaglanden MC, the Hague;

Department of Neurology[15], Radiology[16], HAGA Hospital, the Hague;

Department of Neurology[17], Radiology[18], University Medical Center Utrecht;

Department of Neurology[19], Neurosurgery[20], Radiology[27], Radboud University Medical Center, Nijmegen;

Department of Neurology[21], Radiology[26], Elisabeth-TweeSteden ziekenhuis, Tilburg;

Department of Neurology[22], Radiology[23], Isala Klinieken, Zwolle;

Department of Neurology[24], Radiology[25], Reinier de Graaf Gasthuis, Delft;

Department of Neurology[28], Radiology[29], University Medical Center Groningen;

Department of Neurology[30], Radiology[31], Atrium Medical Center, Heerlen;

Department of Neurology[32], Radiology[33], Catharina Hospital, Eindhoven;

Department of Neurology[34], Radiology[35], Medisch Spectrum Twente, Enschede, (currently Deventer Hospital[47]);

Department of Radiology[36], Amsterdam UMC, Vrije Universiteit van Amsterdam, Amsterdam;

Department of Radiology[37], Noordwest Ziekenhuisgroep, Alkmaar;

Department of Radiology[38], Texas Stroke Institute, Texas, United States of America;

Department of Neurology[42], Radiology[41], Albert Schweitzer Hospital, Dordrecht;

Department of Neurology[43], Radiology[46], Amphia Hospital, Breda.
